# Applying an extended theoretical framework for data collection mode to health services research

**DOI:** 10.1186/1472-6963-10-180

**Published:** 2010-06-24

**Authors:** Michael R Robling, David K Ingledew, Giles Greene, Adrian Sayers, Chris Shaw, Lesley Sander, Ian T Russell, John G Williams, Kerenza Hood

**Affiliations:** 1 South East Wales Trials Unit, School of Medicine, Cardiff University, Neuadd Meirionydd, Heath Park, Cardiff, Wales, CF14 4YS, UK; 2 School of Psychology, Bangor University, Bangor, Wales, LL57 2DG, UK; 3 Department of Primary Care & Public Health, School of Medicine, Cardiff University, Neuadd Meirionydd, Heath Park, Cardiff, Wales, CF14 4YS, UK; 4 Academic Rheumatology, University of Bristol, Avon Orthopaedic Centre, Southmead Hospital, Bristol, England, BS10 5NB, UK; 5 Faculty of Health, Sport & Science, University of Glamorgan, Pontypridd, Wales, CF37 1DL, UK; 6 Support Unit for Research Evidence, Cardiff University, Heath Park Campus, Cardiff, Wales, CF14 4XN, UK; 7 School of Medicine, Swansea University Singleton Park, Swansea, Wales, SA2 8PP, UK

## Abstract

**Background:**

Over the last 30 years options for collecting self-reported data in health surveys and questionnaires have increased with technological advances. However, mode of data collection such as face-to-face interview or telephone interview can affect how individuals respond to questionnaires. This paper adapts a framework for understanding mode effects on response quality and applies it to a health research context.

**Discussion:**

Data collection modes are distinguished by key features (whether the survey is self- or interviewer-administered, whether or not it is conducted by telephone, whether or not it is computerised, whether it is presented visually or aurally). Psychological appraisal of the survey request will initially entail factors such as the cognitive burden upon the respondent as well as more general considerations about participation. Subsequent psychological response processes will further determine how features of the data collection mode impact upon the quality of response provided. Additional antecedent factors which may further interact with the response generation process are also discussed. These include features of the construct being measured such as sensitivity, and of the respondent themselves (e.g. their socio-demographic characteristics). How features of this framework relate to health research is illustrated by example.

**Summary:**

Mode features can affect response quality. Much existing evidence has a broad social sciences research base but is of importance to health research. Approaches to managing mode feature effects are discussed. Greater consideration must be given to how features of different data collection approaches affect response from participants in studies. Study reports should better clarify such features rather than rely upon global descriptions of data collection mode.

## Background

Understanding the unique experience of both users and providers of health services requires a broad range of suitably robust qualitative and quantitative methods. Both observational (e.g. epidemiological cohort) and interventional (e.g. randomised controlled trials) studies may collect data in a variety of ways, and often require self-report from study participants. Increasingly in clinical studies, clinical indicators and outcomes will form part of an assessment package where patient lifestyle choices and behaviour, attitudes and satisfaction with healthcare provision are a major focus. Health researchers need to both be re-assured and provide re-assurance that the measurement tools available are fit-for-purpose across a wide range of contexts. This applies not only to the survey instrument itself, but also to the way it is delivered and responded to by the participant.

Options for collecting quantitative self-report data have expanded substantially over the last 30 years, stimulated by technological advances in telephony and computing. The advent of remote data capture has led to the possibility of clinical trials being conducted over the internet [1,2]. Concerns about survey non-response rates have also led researchers to innovate - resulting in greater diversity in data collection [3]. Consequently, otherwise comparable studies may use different methods of data collection. Similarly, a single study using a sequential mixed mode design may involve, for example, baseline data collection by self-completion questionnaire and follow-up by telephone interview. This has led to questions about the comparability of data collected using different methods [4].

This article applies a conceptual framework to examine the differences generated by the use of different modes of data collection. Whilst there is considerable evidence about the effect of different data collection modes upon response rates, the article addresses the processes that may ultimately impact upon response quality [5-8]. The framework draws upon an existing cognitive model of survey response by Tourangeau and colleagues which addresses how the impact of different data collection modes may be mediated by key variables [9]. Furthermore, the article extends the focus of the model to highlight specific psychological response processes that may follow initial appraisal of survey stimulus. Whilst much of the empirical evidence for mode effects has been generated by research in other sectors, the relevance for health research will be explored. In doing so, other mediators of response will be highlighted.

It is important to clarify what lies outside the scope of the current review. Whilst mode of data collection can impact upon response *rate *as well as response *content*, that is not the focus of this paper. Similarly, approaches that integrate modes of data collection within a study or synthesise data collected by varying modes across studies are only addressed in passing. Although these are important issues for health researchers, this review concentrates on how mode of data collection affects the nature of the response provided by respondents with a particular emphasis on research within the health sciences.

Variance attributable to measurement method rather than the intended construct being measured has been well recognised in the psychological literature and includes biases such as social desirability and acquiescence bias [10]. This narrative review has been developed alongside an ongoing systematic literature review of mode effects in self-reported subjective outcomes [11]. The article highlights for researchers how different methods of collecting self-reported health data may introduce bias and how features of the context of data collection in a health setting such as patient role may modify such effects.

## Discussion

### Modes and mode features

#### What are modes?

Early options for survey data collection were either face-to-face interview, mail, or telephone. Evolution within each of these three modes led to developments such as computer-assisted personal interview (CAPI), web-delivered surveys and interactive voice response (IVR) (see glossary in table 1). Web-based and wireless technologies such as mobile and PDA-(Personal Digital Assistant) based telephony have further stimulated the development of data collection methods and offer greater efficiency compared to traditional data collection methods such paper-based face-to-face interviews [12]. Within and across each mode a range of options are now available and are likely to continue expanding.

**Table 1:** Glossary of common acronyms and technical words

Acronym	
ACASI	Audio Computer Assisted Self-Interview
Acquiescence	A response bias whereby respondents simply agree with an attitudinal statements regardless of content
CAPI	Computer Assisted Personal Interview
CAT	Computerised Adaptive Testing
CATI	Computer Assisted Telephone Interview
IRT	Item Response Theory
IVR	Interactive Voice Response
Optimising	The process of carefully and comprehensively proceeding through all cognitive steps required when answering a survey question.
PAPI	Paper And Pencil Interview
PDA	Personal Digital Assistant (handheld computer)
PROM	Patient reported outcome measure
Satisficing	A strategy of providing a satisfactory response to a survey question without the respondent expending the intended cognitive effort. This may be due to either incomplete, biased or absent retrieval and/or integration of information when responding.

A recent example of technologically enabled mode development is computerised adaptive testing (CAT). Approaches such as item response theory (IRT) modelling allow for survey respondents to receive differing sets of calibrated question items when measuring a common underlying construct (like health-related quality of life) [13]. Combined with technological advances, this allows for efficient individualised patient surveys though the use of computerised adaptive testing [14]. In clinical populations, CAT may reduce response burden, increase sensitivity to clinically important changes and provide greater precision (reducing sample size requirements) [13]. Although IRT-driven CAT may be less beneficial where symptoms are being assessed by single survey items, more general computer-aided testing which mimics the normal clinical interview may be successfully used in combination with IRT-based CAT [15].

#### What are the key features of different data collection modes?

The choice of mode has natural consequences for how questions are worded. Face-to-face interviews, for example, may use longer and more complex items, more adjectival scale descriptors and show cards [16]. In contrast, telephone interviews are more likely to have shorter scales, use only endpoint descriptors and are less able to use show cards. However, even when consistent question wording is maintained across modes there will still be variation in how the survey approach is appraised psychologically by respondents.

The inherent complexity of any one data collection approach (e.g. the individual characteristics of a single face-to-face interview paper-based survey) and increasing technological innovation means that trying to categorise all approaches as one or other mode may be too simplistic. Attention has therefore been focused upon survey design features that might influence response. Two recent models by Groves and by Tourangeau illustrate this [7,9]. Tourangeau identified five features: how respondents were contacted; the presentational medium (e.g. paper or electronic); method of administration (interviewer- or self-administered); sensory input channel; and response mode [9]. Groves also distinguished five features: degree of interviewer involvement, level of interaction with respondent, degree of privacy, channels of communication (i.e. sensory modalities), degree of technology [7]. Whilst both models cover similar ground, Groves places a greater emphasis upon the nature of the relationship between respondent and interviewer. Both models attempt to isolate the active ingredients of survey mode. However, Groves and colleagues note that in practice differing combinations of features make generalisation difficult - reflected in their emphasis upon each individual feature being represented as a continuum. Although research on data collection methods has traditionally referred to mode, given the complexity highlighted above where appropriate we use the term mode feature in this article.

### How mode features influence response quality

Common to several information processing models of how respondents answer survey questions are four basic stages: comprehension of the question; retrieval of information from autobiographical memory; use of heuristic and decision processes to estimate an answer; and response formulation [17]. At each stage, a respondent may employ certain processes when answering a question which may result in response error.

Of the features that might vary across data collection method, Tourangeau and others proposed four which may be particularly influential in affecting response: whether a survey schedule is self- or interviewer-administered, the use of a telephone, computerisation, and whether survey items are read by (or to) the respondent [9]. Although this article focuses on differences between these broad mode features, there may still be considerable heterogeneity within each. For example, computerisation in the form of an individual web-delivered survey may apparently provide a standardised stimulus (i.e. overall package of features) to the respondent, but different hardware and software configurations for each user may violate this assumption [12].

Tourangeau considered three variables to *mediate *the impact of mode feature: degree of impersonality, the sense of legitimacy engendered by the survey approach and level of cognitive burden imposed. Both impersonality and legitimacy represent the respondent's perceptions of the survey approach and instrument. Cognitive burden, impersonality and legitimacy are a function of the interaction between data collection method and individual respondent (and subject to individual variation). Nevertheless, the level of cognitive burden experienced by individuals is less dependent upon the respondent's psychological appraisal of the survey task than perceptions of either impersonality or legitimacy.

**Figure 1:**
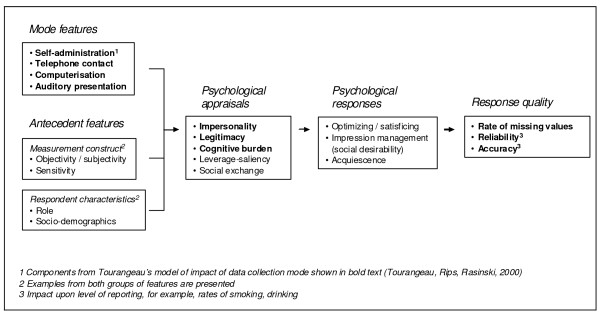
**Mode features and other antecedent features influencing response quality**.

The relationships between these mode features, mediating variables, and three response quality indicators (rate of missing values, reliability, accuracy) are shown in figure 1 and have been previously described by Tourangeau and colleagues [9]. In this article, we further distinguish between psychological appraisals and psychological responses. Psychological appraisals entail initial processing of salient features by individual respondents and incorporate the mediators described by Tourangeau. Two additional appraisal processes are included (Leverage-saliency and Social Exchange) and are described below. Initial appraisal then moves onto psychological response processes. In this amended model, these processes include Optimising/Satisficing, Impression management and Acquiescence [18]. Each of these processes is described below and together they represent differing theoretical explanations for an individual's response. The extent to which they are distinct or related processes is also examined.

Other features may also modify response and are added to the article framework. They include features of the Respondent (the information provider) and Construct (what is being measured). These features are not directly related to the method of data collection. Some of these features are implied by the mediators described by Tourangeau (e.g. the sensitivity of the construct is implicit to the importance of Impersonality). Nevertheless, we consider it important to separate out these features in this framework. Examples of both sets of features are provided but are intended to be indicative rather than exhaustive listings. Finally, whilst there may be no unique feature to distinguish between data collection in health and other research contexts, we have used where we can examples of particular relevance to health.

#### How are data collection stimuli appraised by respondents?

##### Impersonality

The need for approval may restrict disclosure of certain information. Static or dynamic cues (derived from an interviewer's physical appearance or behaviour) provide a social context which may affect interaction [19]. Self-administration provides privacy during data collection. Thus, Jones and Forrest found greater rates of reported abortion amongst women using self-administration methods compared to a personal interview [20]. People may experience a greater degree of privacy when interacting with a computer and feel that computer-administered assessments are more anonymous [21].

The greater expected privacy for methods such as ACASI (audio computer assisted self-interview) has been associated with increased reporting of sensitive and stigmatising behaviours [22]. It is therefore possible that humanising a computerised data collection interface (for example, the use of visual images of researchers within computerised forms) could increase mis-reporting [23]. For example, Sproull and colleagues found higher social desirability scores amongst respondents to a human-like computer interface compared to a text-based interface [24]. However, others have found little support for this effect in social surveys [23]. Certain data collection methods may be introduced specifically to address privacy concerns - for example, interactive voice response and telephone ACASI. However, there is also evidence that computers may reduce feelings of privacy [25]. The need for privacy will vary with the sensitivity of survey topic. Whilst Smith found the impact of others in response to the US General Social Survey to be mostly negligible, some significant effects were found [26]. For example, respondents rated their health less positively when reporting in the presence of others compared to lone respondents.

##### Legitimacy

Some methods restrict opportunities for establishing researcher credentials, for example, when there is no interviewer physically present. A respondent's perception of survey legitimacy could also be enhanced, albeit unintentionally, by the use of computers. Whilst this may be only a transient phenomenon as computers become more familiar as data collection tools, other technological advances may produce similar effects (for example, PDAs).

##### Cognitive burden

Burden may be influenced by self-administration, level of computerisation and channel of presentation. Survey design that broadly accommodates the natural processes of responding to questions across these features is likely to be less prone to error.

##### Leverage-saliency theory

This general model of *survey participation *was proposed by Groves and colleagues and evaluates the balance of various attributes contributing to a decision to participate in a survey [27]. Each attribute (for example, a financial incentive) varies in importance (leverage) and momentary salience to an individual. Both leverage and salience may vary with the method of data collection and interact with other attributes of the survey (for example, item sensitivity). Thus, face-to-face interviewers may be able to convey greater salience to responders through tailoring their initial encounter. This common thread of the presence of an interviewer may enhance the perceived importance of the survey to a respondent, which may first increase their likelihood of participating (response rate) and second enhance perceived legitimacy (response quality). The former effect - participation decisions alone - is not examined further in this review. It is possible that the latter effect of mode feature on response quality may be particularly important in clinical studies if data are being collected by face-to-face interview with a research nurse (for example) rather than by mailed questionnaire.

##### Social exchange theory

This theory views the probability of an action being completed as dependent upon an individual's perception of the rewards gained and the costs incurred in complying, and their trust in the researcher. Dillman applied the theory to explaining response to survey requests - mostly in terms of response rate, rather than quality [28]. However, he noted how switching between different modes within a single survey may allow greater opportunities for communicating greater rewards, lowering costs and increasing trust. This focus upon rewards may become increasingly important as response rates in general become more difficult to maintain. Furthermore, the use of a sequential mixed mode design for non-respondent follow-up within a survey may enhance perceptions of the importance of the research itself by virtue of the researcher's continued effort.

Unlike the first three appraisal processes described above, both leverage-saliency and social exchange address broader participation decisions. Features of different data collection modes may affect such decision-making for example, through perceived legitimacy. Other features in the framework considered to modify response may also influence participation decisions according to these theories (e.g. the sensitivity of the construct being measured).

#### Explaining mode feature effects: psychological responses following appraisal

Initial appraisal of survey stimulus will result in a response process which further mediates response quality. Several explanatory psychological theories have been proposed. We focus upon three general theories of response formulation (optimizing/satisficing, social desirability and acquiescence).

##### 'Taking the easy way out' - Optimizing and satisficing

Krosnick described optimizing and satisficing as two ends of a continuum of thoroughness of the response process [18,29]. Full engagement in survey response represents the ideal response strategy (optimizing) in contrast to incomplete engagement (satisficing). The theory acknowledges the cognitive complexity of survey responding. A respondent may proceed through each cognitive step less diligently when providing a survey response or they may omit *information retrieval *and *judgement *completely (examples of weak and strong satisficing respectively). In either situation, respondents may use a variety of decision heuristics when responding. Three factors are considered to influence the likelihood of satisficing: respondent ability, respondent motivation and task difficulty [18,30]. Krosnick defines respondent ability (or cognitive sophistication) as the ability to retrieve information from memory and integrate it into verbally expressed judgements [18]. Optimizing occurs when respondents have sufficient cognitive sophistication to process the request, when they are sufficiently motivated, and when the task requirements are minimal [31].

Mode feature effects may influence optimizing through differences in non-verbal communication, interview pace (speed) and multitasking. First, the enthusiastic non-verbal behaviour of an interviewer may stimulate and maintain respondent motivation. Experienced interviewers react to non-verbal cues (for example, expressions relating to disinterest) and respond appropriately. Such advantages are lost in a telephone interview with interviewers relying on changes in verbal tones to judge respondent engagement. Whilst the role of an interviewer to enhance the legitimacy of the survey request was highlighted in Tourangeau's framework, this additional motivation and support function was not clarified [9]. Second, interview pace may differ between telephone and face-to-face contact, in part because silent pauses are less comfortable on the telephone. A faster pace by the interviewer may increase the task difficulty (cognitive burden) and encourage the respondent to take less effort when formulating their response. Pace can vary between self- and interviewer-administered methods. A mailed questionnaire may be completed at the respondent's own pace allowing them greater understanding of survey questions compared to interviewer-driven methods. Tourangeau and colleagues omitted pace as a mediating variable from their model of mode effects because they considered insufficient evidence has accrued to support its role [9]. Interview pace has been suggested as an explanation for observed results but the effects of pace have not necessarily been tested independently from other mode features (for example, see Kelly and colleagues, 2008) [32]. Nevertheless, it is discussed here due to its hypothesised effect [18]. Finally, distraction due to respondent multitasking may be more likely in telephone interviews compared to face-to-face interviews (e.g. telephone respondents continuing to interact with family members, conduct household task whilst on the telephone). Such distraction increases task difficulty and thus may promote satisficing [18].

Optimizing/Satisficing has been used to explain a variety of survey phenomena, for example, response order effects (where changes in response distributions result from changes in the presentational order of response options) [33]. Visual presentation of survey questions with categorical response options may allow greater time for processing initial options leading to primacy effects in those inclined to satisfice. Weak satisficing may also result from the termination of evaluative processing (of a list of response options) when a reasonable response option has been encountered. This may occur for response to items with adjectival response scales and also for ranking tasks [18]. In contrast, aural presentation of items may cause respondents to devote more effort to processing later response options (which remain in short-term memory after an interviewer pauses) - leading to recency effects in satisficing respondents [18]. Telephone interviews can increase satisficing (and social desirability response bias) compared to face-to-face interviews [31]. An example of a theoretically driven experimental study that has applied this parsimonious model to studying mode feature effects is provided by Jäckle and colleagues [34]. In the setting of an interviewer-delivered social survey, they evaluated the impact of question stimulus (with or without showcards) and the physical presence or absence of interviewer (face-to-face or telephone). In this instance, detected mode feature effects were not attributable to satisficing but to social desirability bias instead.

##### Social desirability

The tendency for individuals to present themselves in a socially desirable manner in the face of sensitive questions has long been inferred from discrepancies between behavioural self-report and documentary evidence. Response effects due to self-presentation are more likely when respondents' behaviour or attitudes differ from their perception of what is socially desirable [35]. This may result in over-reporting of some behaviours and under-reporting of others. Behavioural topics considered to induce over-reporting include being a good citizen, and being well-informed and cultured [36]. Under-reporting may occur with certain illnesses (e.g. cancer and mental ill-health), illegal and non-normative behaviours and financial status. An important distinction has been made between intentional impression management (a conscious attempt to deceive) and unintentional self-deception (where the respondent is unaware of their behaviour) [37]. The former has been found to vary according to whether responses were public or anonymous whilst the latter was invariant across conditions.

Most existing data syntheses of mode feature effects relate to social desirability bias. Sudman and Bradburn found a large difference between telephone- or self-administered surveys compared to face-to-face interviews [35]. Differences in social desirability between mode features have been the subject of subsequent meta-analyses by de Leeuw; Richman and colleagues; and Dwight & Feigelson [38-40].

It is worth noting that Sudman and Bradburn developed a comprehensive coding scheme for their review which was later extended in de Leeuw's work. Difficulties in coding variables with their respective frameworks were noted by Sudman and Bradburn and by Richman and colleagues and is a ubiquitous problem. A greater theoretically-driven emphasis upon mode features should aid both future empirical research and also such data syntheses. The Richman review is particularly notable for its attempt to test explicit a priori hypotheses, its operational definition of sensitivity and its focus upon features rather than overarching modes.

Collectively, these reviews provide support for the importance of self-administration and consequently impersonality. Richman and colleagues concluded that there was no overall difference between computer and paper-and-pencil scales. This is consistent with Tourangeau's original model which directly links computerisation to legitimacy and cognitive burden but not to impersonality. From Sudman and Bradburn's review, and from de Leeuw's subsequent review it is clear that other factors may significantly modify the relationship between mode feature and social desirability bias. For example, Whitener and Klein found a significant interaction between the respondent's social environment at the time of data collection (individual vs group) and method of administration [41]. Similarly, Richman and colleagues found that year of publication was a significant effect modifier when comparing, for example, computer and paper and pencil questionnaires [39]. The authors suggested that the decreasing mode effect observed over time may result from hardware and software innovations which have increasingly minimised presentational differences between these modes. Future exploration of mode effects should therefore, be mindful of such potential effect changes over time.

##### Acquiescence

Asking respondents to agree or disagree with attitudinal statements may be associated with acquiescence - respondents agreeing with items regardless of their content [42]. Acquiescence may result from respondents taking shortcuts in the response process and paying only superficial attention to interview cues [7]. Knowles and Condon categorise meta-theoretical approaches to acquiescence as either addressing motivational or cognitive aspects of the response process [43]. Krosnick suggested that acquiescence may be explained by the notion of satisficing due to either cognitive or motivational factors [29]. Thus, the role of mode features in varying impersonality and cognitive burden as described above would seem equally applicable here.

There is mixed evidence for a mode feature effect for acquiescence. De Leeuw reported no difference in acquiescence between mail, face-to-face and telephone interviews [38]. However, Jordan and colleagues found greater acquiescence bias in telephone interviews compared to face-to-face interviews [44]. Holbrook and colleagues also found greater acquiescence amongst telephone respondents compared to face-to-face respondents in two separate surveys [31].

### What are the consequences of mode feature effects for response quality?

Several mode feature effects on response quality are listed in figure 1 and include rate of *missing data *[45]. Computerisation and using an interviewer will decrease missing data due to unintentional skipping. Intentional skipping may also occur and be affected by both the impersonality afforded the respondent and the legitimacy of the survey approach. The model of Tourangeau and colleagues describes how the *reliability *of self-reported data may be affected by the cognitive burden placed upon the respondent [9]. De Leeuw provides a good illustration of how the internal consistency (psychometric reliability) of summary scales may be varied by mode features through (i) differences in interview pace and (ii) the opportunity for respondents to relate their responses to scale items to each other [38]. The reliability of both multiple and single item measures across surveys (and across waves of data collection) may also be affected by any mode feature effects resulting from the psychological appraisal and response processes described above.

Tourangeau and colleagues highlight how *accuracy *(validity) of the data may be affected by impersonality and legitimacy. Both unreliable and inaccurate reporting will be represented by variations in the *level *of an attribute being reported. For example, a consequence of socially desirable responding will be under- or over-reporting of attitudes and behaviour. This may vary depending upon the degree of impersonality and perceived legitimacy.

### Additional antecedent features

Two further sets of variables are considered in the article framework presented in figure 1, Measurement construct and Respondent characteristics. Both represent antecedent features which may further interact with the response process described. For the purposes of this article, they will be described particularly in relation to health research.

#### Measurement construct

##### Objective/subjective constructs

Constructs being measured will vary according to whether they are subjective or objectively verifiable. Health-related quality of life and health status are increasingly assessed using standardised self-report measures (increasingly referred to as Patient Reported Outcome Measures or PROMs in the health domain). Whilst the construct being assessed by such measures may in some cases be externally verified (e.g. observation of physical function) for other constructs (e.g. pain) this may not be possible. Furthermore, the subjective perspective of the individual may be an intrinsic component of the construct being measured [46,47]. Cote and Buckley reviewed 64 construct validation studies from a range of disciplines (marketing, psychology/sociology, other business, education) and found 40% of observed variance in attitudes (subjective variable) was due to method (i.e. the influence of measurement instrument), compared to 30% due to the trait itself [48]. For more objective constructs variance due to method was lower indicating the particular challenge for assessing subjective constructs.

##### Sensitivity

Certain clinical topics are more likely to induce social desirability response bias - potentially accentuating mode feature effects. Such topics include sensitive clinical conditions (for example, HIV status) and health-related behaviours (for example, smoking). An illustrative example is provide by Ghanem and colleagues who found more frequent self-reports of sensitive sexual behaviours (e.g. number of sexual partners in preceding month) using ACASI compared to face to face interview amongst attendees of a public sexually transmitted diseases clinic [49].

#### Respondent characteristics

##### Respondent role

In much of the research contributing to meta-analyses of mode effects on social desirability, the outcome of the assessment was not personally important for study subjects (for example, participants being undergraduate students) [39]. Further methodological research in applied rather than laboratory settings will help determine whether mode feature effects are generalisable to wider populations of respondents. It is possible that the motivations of patients (e.g. perceived personal gain and perceived benefits) will reflect their clinical circumstances as well as other personality characteristics [50-52]. It is therefore worth investigating whether self-perceived clinical need (for example) may be a more potent driver of biased responding than social desirability and whether this modifies mode feature effects.

In a review of satisfaction with healthcare, the location of data collection was found to moderate the level of satisfaction reported, with on site surveys generating less critical response [8]. Crow and colleagues noted how the likelihood of providing socially desirable responses was commonly linked by authors to the degree of impersonality afforded when collecting data either on or off-site.

Another role consideration involves the relationship between respondent and researcher. The relationship between patient and healthcare professional may be more influential than that between social survey respondent and researcher. A survey request may be viewed as particularly legitimate in the former case, and less so in the latter [51]. Response bias due to satisficing may be less of a problem in such clinical populations than in non-clinical populations. Systematic evaluation of the consequence of respondent role in modifying mode feature effects warrants further research.

##### Respondent sociodemographics

There is some indication of differential mode feature effects across demographic characteristics. For example, Hewitt reports variation in sexual activity reporting between modes (audio-CASI and personal interview) by age, ethnicity, educational attainment and income [53]. The epidemiology of different clinical conditions will be reflected by patient populations that have certain characteristics, for example, being older. This may have consequences for cognitive burden or perceptions of legitimacy in particular health studies.

### Particular issues in health research

In considering modes and mode feature effects we will focus upon three issues that may be of particular relevance to those collecting data in a health context: antecedent features, constraints in choice of mode and the use to which the data is being put.

#### Particular antecedent features

Certain antecedent conditions and aspects of the construct being measured may be particularly relevant in health-related studies. Consider the example of quality of life assessment in clinical trials of palliative care patients from the perspective of response optimising. Motivation to respond may be high, but may be compromised by an advanced state of illness. Using a skilled interviewer may increase the likelihood of optimizing over an approach offering no such opportunity to motivate and assist the patient. Physical ability to respond (for example, verbally or via a keyboard) may be substantially impaired. This may affect response completeness but if the overall response burden (including cognitive burden) is increased it may also lead to satisficing. In practice, choice of data collection method will be driven as much by ethical considerations about what is acceptable for vulnerable respondents.

Are there features of self-reported data collection in health that are particularly different from other settings of relevance to mode feature effects? Surveys will be applied in health research in a wide variety of ways, and some will be indistinguishable in method from some social surveys (e.g. epidemiological sample surveys). Some contexts for data collection in health research may though be very different from elsewhere. Data collection in randomised controlled trials of therapeutic interventions may often include patient-reported outcome measures to assess differences in outcome. How antecedent features in the trial - in particular those associated with respondent role - may influence psychological appraisal and response is hypothesised in table 2. These antecedent characteristics may potentially either promote or reduce the adverse impact of mode feature effects. The extent to which these effects may be present will need further research, and at least would require consideration in trial design.

**Table 2:** How mode and antecedent features may influence response: the example of respondent role in a clinical trial

Antecedent features in trial	Appraisal and response: some research hypotheses
*Respondent role: *Participants approached for participation by their professional carer	*Legitimacy: *An established patient-carer relationship with high levels of regard for the researcher may enhance legitimacy of survey request sufficiently to modify mode feature effects and therefore reduce satisficing
*Respondent role: *Participants are consented through formally documented process	*Legitimacy: *The formality and detail of consenting process may enhance legitimacy of survey request sufficiently to modify mode feature effects and therefore reduce satisficing
*Respondent role: *Participants provide self-reported data at the site of delivery for their healthcare	*Impersonality: *On-site data collection may increase need for confidential and anonymous reporting sufficiently to promote adverse effects of mode feature effects and introduce social desirability bias
*Respondent role/sensitivity: *Participants are patients with an on-going clinical need	*Cognitive burden: *Health status of respondent may increase overall cognitive burden to modify mode feature effects and increase satisficing. Burden and therefore, effects may vary with disease and treatment progression. *Impersonality: *The nature of the condition may increase the need for confidential and anonymous reporting sufficiently to promote adverse mode feature effects and introduce social desirability bias.
*Respondent role: *Participants are patients in receipt of therapeutic intervention	*Legitimacy/leverage-saliency: *The requirement for treatment and the opportunity for novel therapy enhance legitimacy and the perceived importance/salience of the research. This may minimise adverse mode feature effects to reduce satisficing.

#### Particular constraints on choice of mode

As in social surveys, mode feature effects will be one of several design considerations when collecting health survey data. Surveying patients introduces ethical and logistical considerations which, in turn, may determine or limit choice of data collection method. Quality criteria such as appropriateness and acceptability may be important design drivers [54]. For example, Dale and Hagen reviewed nine studies comparing PDAs with pen-and-paper methods and found higher levels of compliance and patient preference with PDAs [55]. Electronic forms of data collection may offer advantages in terms of speed of completion, decreasing patient burden and enhancing acceptability [56,57]. The appropriateness of different data collection modes may vary by patient group - for example, with impaired response ability due to sensory loss [58]. Health researchers need to balance a consideration of mode feature effects with other possible mode constraints when making decisions about data collection methods.

#### Particular uses of data

Evaluating mode feature effects will be particularly important as survey instruments start to play a bigger role in the provision of clinical care, rather than solely in research. Patient reported outcome measures are increasingly being applied and evaluated in routine clinical practice [59-61]. Benefits have been found in improving process of care but there is less consistent evidence for impact on health status [59,62-64].

Perceived benefits of using such patient reported outcomes include assessing the impact on patients of healthcare interventions, guiding resource allocation, and enhancing clinical governance [60]. Computerised data collection may be especially important if results are to inform actual consultations, but would require suitably supported technology to permit this [65,66]. With only mixed evidence of clinical benefit, Guyatt and colleagues highlight computerised-based methods of collecting subjective data in clinical practice as a lower cost approach [64].

In this clinical service context, psychological responses such as social desirability bias may vary according to whether patient data is being collected to inform treatment decision-making or clinical audit. Method of data collection may similarly play a role in varying the quality of response provided. However, routinely using subjective outcome measures in clinical practice will require a clear theoretical basis for their use and implementation, may require additional training and support for health professionals, and investment in the technology to support its effective implementation which is preferably cost neutral [67-70] Overall though, it may be that any biasing effect of mode feature may be less salient in situations where information is being used as part of a consultation to guide management, and may be more so where data is being collected routinely across organisational boundaries as part of clinical audit or governance.

### Managing mode feature effects in health

Managing mode feature effects requires identification of their potential impact. This paper has focused upon response quality as one source of error in data collection. Two other sources of error influenced by mode are coverage error and non-response error [71]. In the former, bias may be introduced if some members of the target population are effectively excluded by features of the chosen mode of data collection. For example, epidemiological surveys using random digit dialling which exclude people without landline telephones may result in biased estimates as households shift to wireless only telephones [72]. Response rates vary by mode of data collection and different population sub-groups vary in the likelihood of responding to different modes [71]. For example, Chittleborough and colleagues found differences by education, employment status and occupation amongst those responding to telephone and face-to-face health surveys in Australia [73].

Social surveys commonly blend different modes of data collection to reduce cost (e.g. by a graduated approach moving from cheaper to more expensive methods [7]). Mixing modes can also maximise response rates by, for example, allowing respondents a choice about how they respond.

In the long term it may prove possible to correct statistically for mode feature effects if consistent patterns emerge from meta-analyses of empirical studies. Alternatively, approaches to reducing socially desirable responding have targeted both the question threat and confidentiality. An example of the latter is the Randomised Response Technique which guarantees privacy [74,75]. Another approach is the use of goal priming (i.e. the manipulation and activation of an individual's own goals to subsequently motivate their behaviour) where respondents are influenced sub-consciously to respond more honestly [76].

#### Evaluating and reporting mode feature effects

As described above, the evaluation of data collection method within individual studies is usually complicated by the package of features representing any one mode. Groves and colleagues described two broad approaches to the evaluation of effects due to mode features [7]. The first and more pragmatic strategy involves assessing a package of features between two or more modes. Such a strategy may not provide a clear explanation for resulting response differences, but may satisfy concerns about whether one broad modal approach may be replaced by another. The second approach attempts to determine the features underlying differences found between two modes. This theoretically driven strategy may become increasingly important as data collection methods continue to evolve and increase in complexity.

As global descriptions of data collection method can obscure underlying mode features, comparative studies should describe these features more fully. This would enable data synthesis, providing greater transparency of method and aid replication [39].

## Summary

This article has considered how features of data collection mode may impact upon response quality and key messages are summarised below. It has added to a model proposed by Tourangeau and colleagues by drawing apart psychological appraisal and response processes in mediating the effect of mode features. It has also considered other antecedent features that might influence response quality. Mode feature response effects have been most thoroughly reviewed empirically in relation to social desirability bias. Overall effects have been small, although evidence of significant effect modifiers emphasises the need to evaluate mode features rather than simply overall mode. A consistent finding across the reviews is the important moderating effect of year of publication for comparisons involving both telephone and computers. Therefore, mode feature comparisons are likely to remain important as new technologies emerge for collecting data. Although much of the empirical research underpinning the reviewed model has been generated within other academic domains, the messages are nonetheless generally applicable to clinical and health research. Future evidence syntheses may confirm or amend the proposed model but this requires as a precursor greater attention to theoretically driven data collection about mode features.

### Key messages

• Choice of data collection mode can introduce measurement error, detrimentally affecting accurate and reliable survey response.

• Surveys in health service and research posses similar features to surveys in other settings.

• Features of the clinical setting, the respondent role and the health survey content may emphasise psychological appraisal and psychological responses implicated in mode feature effects.

• The extent to which these features of health surveys result in consistent mode effects which are different from other survey context requires further evaluation.

• Evaluation of mode effects should identify and report key features of data collection method, not simply categorise by overall mode.

• Mode feature effects are primarily important when data collected via different modes are combined for analysis or interpretation. Evidence for consistent mode effects may nevertheless permit routine adjustment to help manage such effects.

## Competing interests

The authors declare that they have no competing interests.

## Authors' contributions

MR and DI have been in charge of developing the main arguments presented in the article, have led initial drafting of the article and written the final manuscript. GG, AS, CS, LS, IR, JW and KH have made substantial contributions to developing the arguments in the article, have critically revised the article for substantive intellectual content and have read and approved the final manuscript.

## Pre-publication history

The pre-publication history for this paper can be accessed here:

http://www.biomedcentral.com/1472-6963/10/180/prepub
